# Patient reported outcome measures in childbirth and postpartum maternal quality of life: a protocol for systematic review of measurement properties.

**DOI:** 10.12688/hrbopenres.13445.1

**Published:** 2021-11-03

**Authors:** Laura J. O'Byrne, Gillian Maher, Ali S. Khashan, Richard Greene, John Browne, Fergus P. McCarthy

**Affiliations:** 1 Department of Obstetrics and Gynaecology, Cork University Maternity Hospital, Wilton, Cork, Ireland; 2 INFANT Research Centre, Cork, Ireland; 3 Department of Epidemiology and Public Health, University College Cork, Cork, Ireland

**Keywords:** Maternity care, Patient reported outcome measure, postpartum, quality of life, quality of care, validity, Reliability, responsiveness

## Abstract

**Background:** Patient centred healthcare is the corner stone to many healthcare strategies. Patient specific health needs should be at the fore of healthcare improvements and quality measurements.  Patient reported outcome measures (PROM) that support real world clinical effectiveness assessments are increasingly being used to highlight domains where there is the greatest scope for change.

**Objectives:** This systematic review aims to identify and evaluate existing patient reported assessment measures/tool(s) that can be used in developing a PROM for postpartum women. We will assess and evaluate their measurement properties in a transparent and structured way in accordance with the COSMIN guidelines.

**Methods:** Methodological guidelines for systematic reviews of PROMs have been developed by the COSMIN initiative and will be followed for this systematic review. A systematic literature review will be performed using PubMed and EMBASE from inception to the present day. Two reviewers independently will judge eligibility, conduct data extraction and assess the methodological quality of each study as per COSMIN guidelines. Inclusion criteria: studies should concern PROM with an aim to evaluate measurement properties in the development or the evaluation of a PROM of interest. Included PROMS will focus upon postpartum women assessing morbidity and quality of care. All peer reviewed studies with an assessment tool designed for patient completion will be considered. Exclusion criteria; abstract, letters and non-peer reviewed publications. Studies will be graded on measurement properties and quality of evidence as laid out by COSMIN. All studies and characteristics eligible for inclusion will be summarised and a recommendation to the most suitable measurement tool(s) will be given.

**Discussion:** We will provide a comprehensive description of all available patient reported assessment tools available for childbirth and postpartum quality of life and recommend based on COSMIN guidelines the most suitable instrument(s) available for use.

## Introduction

Patient-centric quality healthcare can only be developed with reliable information. Patient reported outcome measures (PROM) provide an insight into the impact that an intervention or therapy has on the patient, this is particularly important when striving for improvements in maternity care. PROM’s support real world clinical effectiveness assessment for different care models and interventions, describe variation across sociodemographic and clinical groups, highlight domains where there is greatest scope for improvement, and detect variation between providers and institutions that cannot be explained by differences in case mix. There are no current means of capturing outcomes from the perspective of women receiving maternity care. The need for patient-reported outcome measures that can be used in maternity care has been gaining attention in the literature over the past decade. The CROWN initiative, an international initiative led by research journal editors which was established to standardise core outcomes reporting in women’s health research has been calling for supporting PROM sets to be developed in tandem to their core outcome sets, but currently no maternity PROMs exist ^1– 3^.

PROMs are a means of assessing the impact that health events, and interventions have had on constructs such as quality of life. It is important to differentiate PROMs from patient-reported experience measures (PREMs). PREMs are assessments of the patients’ experience of the care they received including whether certain standards of care were met, and their subjective satisfaction with care. There are multiple maternity-specific PREMs in Ireland and the UK: the Irish national maternity experience survey ^4^ and two in the UK, the national maternity services survey ^5^ and ‘You and Your Baby’ survey ^6^.

The ideal PROM should have validity, reliability and be able to measure change over time ^3^. Recent systematic reviews ascertained there are no PROMs currently suitable for a maternity system, but many previously validated tools could be considered when designing a specific PROM for pregnancy and childbirth ^2, 7, 8^. The development of a maternity PROM that covers antenatal, intra-partum, postpartum and neonatal care is challenging. There have been studies recently focusing on measures of recovery and recovery post Caesarean Section ^9, 10^. This project will focus on the generation of a maternity PROM focusing on delivery outcomes which can be applied to all women postpartum. Postpartum quality of life (QoL) will be examined across multiple domains. This includes, but is not limited to the conceptual model of patient outcomes by Wilson and Cleary ^11^; symptom status → functional status → general health perception → overall QoL. This will allow new insights into women’s perspectives of the healthcare they have received and ultimately driving the service into a more patient centred response.

The COnsensus-based Standards for the selection of health Measurement INstruments initiative has improved the selection and assessment of outcome measurement instruments. The COSMIN guidelines will allows us to select the best outcome measurements in a methodological and consistent way ^12^.

The aim of this review is to identify potential tool(s)/measurement that can be used to produce a PROM for postpartum women who have been given care within a maternity system.

## Methods

### Protocol and registration

The present paper is reported in accordance with the recommendations from the Preferred Reporting Items for Systematic Reviews and Meta-Analysis Protocols (PRISMA-P) statement ^13^ (see extended data) ^14^. The review will follow COSMIN guidelines for systematic reviews ^12^. This protocol has been submitted for registration with PROSPERO (submission number 283472). Details of the PROSPERO protocol registration will be provided in the final systematic review.

### Search strategy

A systematic literature review to examine all available objective tools for patient assessment of QoL and health assessment after hospital based maternity care will be performed using the following bibliographic databases: PubMed and EMBASE. Databases will be searched from inception to present and there will be no language restrictions set. Additionally, hand searching of the reference lists of the studies included and key articles on this topic will be searched.

Using the comprehensive PROM filter developed for COSMIN ^15^ and with the assistance of a librarian a search thread was constructed as follows in order to identify all relevant publications (see extended data) ^16^. The search thread included construct search, population search with a measurement properties filter and exclusion filter as recommended by COSMIN. We chose to not included a ‘type of instrument’ in our search strategy as it may possibly exclude potential tools of interest.

Selection of abstracts and full-text articles will be performed by two reviewers independently using Rayyan software. If there is a study that has been deemed relevant by at least one reviewer the full -text article will be retrieved and screened. If a consensus cannot be reached a third senior review will consult.

### Eligibility criteria

The eligibility criteria are in agreement with the COSMIN guidelines for systematic review, namely the PROM(s) should aim to measure the construct of interest QoL. The study sample should represent the population of interest, in our case postpartum women after maternity care. The study should concern PROMS and finally the aim should be the evaluation of one or more measurement properties, the development of a prom or the evaluation of the interpretability of the PROM of interest.

Following the PICO (population, intervention, comparator and outcome) framework our population is postpartum women, Intervention is childbirth, comparator is not required but may include differing modes of birth, Neonatal Intensive Care Unit admission, morbidity and outcome is quality of care. All peer reviewed studies with an assessment tool designed for patient completion will be considered.

Exclusion criteria: abstract, letters and non-peer reviewed publications.

COSMIN recommend the exclusion of studies that only use the PROM as an outcome measurement instrument or in studies in which the PROM is used in a validation study of another instrument. We will examine the possibility of excluding these studies from our search if there are other appropriate tools available. We may consider the inclusion of these studies if there are limited.

### Data extraction

**Figure 1.**
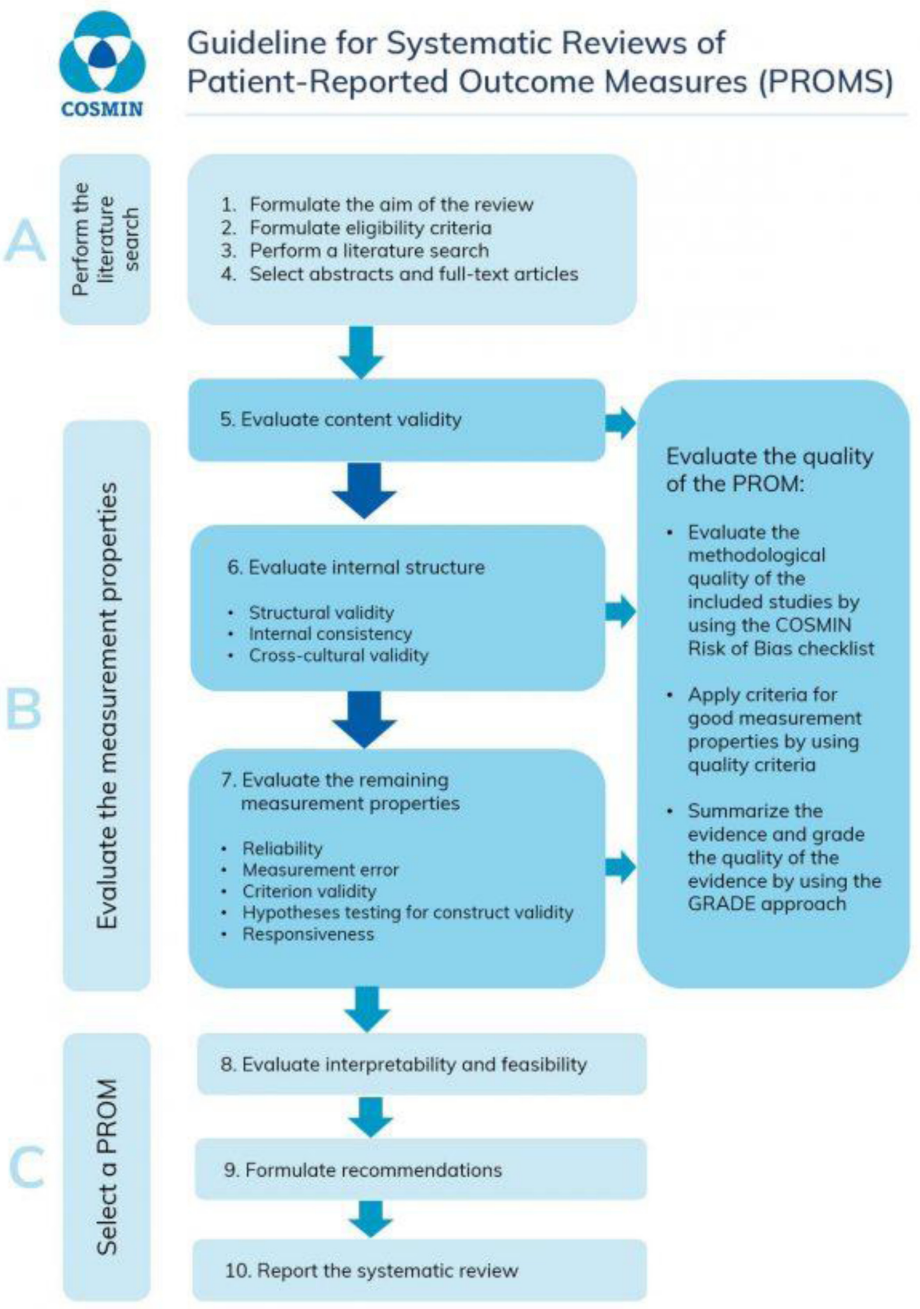
The 10 steps for conducting a systematic review from the COSMIN manual for systematic reviews of patient reported outcome measures (PROMs) ^12^.

PRISMA-P guidelines and COSMIN guidelines for systematic reviews will be followed with the following steps being used to evaluate potential PROM ^17^. These are a ten-step process as described in Figure 1.

The data to be extracted as recommended by the COSMIN systematic review guidelines ^12^ include the characteristics of the tool, characteristics about the population assessed, results on the measurement properties and information on the interpretability and feasibility if available. This information will be extracted independently and presented in an overview table. If any information is missing the reviewers will contact the author for additional information.

Each study will we rated as very good, adequate, doubtful or inadequate quality for the methodology used to assess the psychometric properties of that study. The overall rating of the quality of each study is determined by taking the lowest rating of any standard in the box (i.e. “the worst score counts” principle). The results of each study on measurement property are rated against the criteria for a good measurement property. Each result is then rated as either sufficient (+), insufficient (-), or indeterminant (?) The results will be summarised using the modified GRADE approach. A modified GRADE approach will be used specifically for evaluating measurement properties in systematic reviews of PROMs, the following four factors should be taken into account: (1) risk of bias (i.e. the methodological quality of the studies), (2) inconsistency (i.e. unexplained inconsistency of results across studies), (3) imprecision (i.e. total sample size of the available studies), and (4) indirectness (i.e. evidence from different populations than the population of interest in the review)

The results on all available measurement properties will be quantitatively pooled or qualitatively summarised and against the criteria for good measurement properties to determine whether an overall measurement property is sufficient (+) insufficient (-) inconsistent (±) or indeterminant (?).

### Generating recommendations for the use of a PROM

PRISMA-P guidelines and COSMIN guidelines for systematic reviews will be followed with the following steps being used to evaluate potential PROM ^17^. These are a ten-step process as described in Figure 1.

The data to be extracted as recommended by the COSMIN systematic review guidelines ^12^ include the characteristics of the tool, characteristics about the population assessed, results on the measurement properties and information on the interpretability and feasibility if available. This Information will be extracted independently and presented in an overview table. If any information is missing the reviewers will contact the author for additional information.

Each study will we rated as very good, adequate, doubtful or inadequate quality for the methodology used to assess the psychometric properties of that study. The overall rating of the quality of each study is determined by taking the lowest rating of any standard in the box (i.e. “the worst score counts” principle). The results of each study on measurement property are rated against the criteria for a good measurement property. Each result is then rated as either sufficient (+), insufficient (-), or indeterminant (?) The results will be summarised using the modified GRADE approach. A modified GRADE approach will be used specifically for evaluating measurement properties in systematic reviews of PROMs, the following four factors should be taken into account: (1) risk of bias (i.e. the methodological quality of the studies), (2) inconsistency (i.e. unexplained inconsistency of results across studies), (3) imprecision (i.e. total sample size of the available studies), and (4) indirectness (i.e. evidence from different populations than the population of interest in the review)

The results on all available measurement properties will be quantitatively pooled or qualitatively summarised and against the criteria for good measurement properties to determine whether an overall measurement property is sufficient (+) insufficient (-) inconsistent (±) or indeterminant (?).

A. PROMs with evidence for sufficient content validity (any level) AND at least low-quality evidence for sufficient internal consistency: recommended for use.
B. PROMs where further validation is needed.
C. PROMs with high-quality evidence for an insufficient measurement property: should not be recommended for use.

### Dissemination of information

Publication in a leading journal.

### Study status

Not started.

## Discussion

A complete systematic assessment of the literature to examine available measurement properties is the first step toward developing a usable postpartum PROM. The COSMIN initiative aims to improve the selection of outcome measurement instruments in research and clinical practice by developing tools for selecting the most suitable instrument for the situation at issue and will be followed throughout this review

Our aim is to recommend the best PROM or of combination of self-reported tool(s)/assessment measures to evaluate maternity care postpartum. Each step of the process will be reported on in a systematic and transparent way accompanied by clear recommendations for the most suitable outcome measurement instrument. 

## Data availability

### Underlying data

No data are associated with this article.

### Extended data

Harvard Dataverse: Search thread: Patient reported outcome measures in childbirth and postpartum maternal quality of life: a protocol for systematic review of measurement properties. https://doi.org/10.7910/DVN/WMHKXG ^16^.

### Reporting guidelines

Harvard Dataverse: PRISMA-P checklist for ‘Patient reported outcome measures in childbirth and postpartum maternal quality of life: a protocol for systematic review of measurement properties’. https://doi.org/10.7910/DVN/IXX1EG ^14^.

Data are available under the terms of the Creative Commons Zero "No rights reserved" data waiver (CC0 1.0 Public domain dedication).
