# Supplementary material for: Patient reported outcome measures in childbirth and postpartum maternal quality of life: a protocol for systematic review of measurement properties
Source: HRB Open Res. 2024 Jun 6;4:117. Originally published 2021 Nov 3. [Version 3] doi: 10.12688/hrbopenres.13445.3 (PMC10545984; doi:10.12688/hrbopenres.13445.3)
Supplement: Peer review history

# Reviewer report: Reviewer response for version 3

**DOI:** 10.21956/hrbopenres.15244.r45810

**Published:** 2025-02-26

**Authors:** Susanna Carmona Cañabate

**Affiliations:** 1 Instituto de Investigación Sanitaria Gregorio Marañón, Instituto de Salud Carlos III, Madrid, Spain

This manuscript describes the protocol for conducting a systematic review of “Patient Reported Outcome Measures” (PROM). According to the authors, “currently no maternity PROM exists.”

 The objective of this study is to identify and evaluate patient-reported assessment tools or measures that could contribute to the development of a PROM for postpartum women. The study will systematically assess the measurement properties of these tools, following the COSMIN guidelines to ensure a rigorous and transparent evaluation.

 The findings from this review will be used to generate recommendations on the most reliable and well-supported PROMs, highlighting those with sufficient evidence and consistency for use in postpartum care. I believe that this protocol can significantly improve maternity services by providing a standardized and evidence-based approach to assessing patient-reported outcomes.

Is the study design appropriate for the research question?

Yes

Is the rationale for, and objectives of, the study clearly described?

Yes

Are sufficient details of the methods provided to allow replication by others?

Yes

Are the datasets clearly presented in a useable and accessible format?

Yes

Reviewer Expertise:

Neuroscience of pregnancy and postpartum, Neuroimaging.

I confirm that I have read this submission and believe that I have an appropriate level of expertise to confirm that it is of an acceptable scientific standard.

# Reviewer report: Reviewer response for version 2

**DOI:** 10.21956/hrbopenres.14957.r36180

**Published:** 2023-10-02

**Authors:** Joanna Leaviss

**Affiliations:** 1 University of Sheffield, Sheffield, UK

The protocol describes planned methods for a systematic review of PROMs for maternity care using COSMIN methodology. The authors state that there are currently no maternity specific PROMs.

 The stated aim is to ‘identify potential tool(s)/measurement that can be used to produce a PROM for postpartum women who have been given care within a maternity system’. This will be used to develop a maternity specific PROM with multiple domains. 

 Review question: This needs further clarification (and see comments on inclusion criteria below). Is the aim of the review to identify tools, or is the aim of the review to evaluate the psychometric properties of tools used to assess various measures of QoL in postpartum women? Is this specific part of the project to develop a new tool, or is this specific part of the project to assess what already exists, to take forward to produce a new tool? The review question should reflect this very specific aim.  

 Search strategy: 3 databases is very much the minimum expected for a systematic review. I would generally expect to see more databases searched, and to include the Cochrane library, plus PsychInfo as this is a review of psychometric properties. More supplementary searching should be included, for example, citation searching. 

 Inclusion criteria: Much more detail is need here. PICO isn’t appropriate as childbirth isn’t an ‘intervention’. You could include it as an ‘exposure’, but as there is really no comparator - a PEO structure would be more relevant. 

 A section on ‘types of study’ would also be informative here. The protocol states that ‘The study should concern PROM and finally the aim should be the evaluation of one or more measurement properties, the development of a prom or the evaluation of the interpretability of the PROM of interest.’ I think further detail is needed on the types of PROM that are of interest. The authors have stated in the abstract that there are no maternity specific PROMs. Presumably these will still be searched for however – recent PROMs may have been published. In addition, more clarification is needed on what type of PROMs are expected to be included in light of the anticipated lack of condition-specific PROMs – i.e. generic PROMs such as EQ5D, SF-36 etc. So will the authors be retrieving studies which include the development/validation of generic tools, but only where they have been applied to maternity-specific populations?

 For ‘outcomes’ – the authors state outcome is quality of care. Do they mean quality of life? However, isn’t the outcome the psychometric properties of the tools that are being used to assess QoL? These properties as described in the COSMIN methodology should be briefly listed e.g. content validity; structural validity; internal consistency etc.

 Finally, will the COSMIN ratings be ratified by subject experts and patients at the end of the process?

Reviewer Expertise:

Evidence synthesis methodology.

I confirm that I have read this submission and believe that I have an appropriate level of expertise to confirm that it is of an acceptable scientific standard, however I have significant reservations, as outlined above.

# Response

**Published:** 2024-04-30

**Authors:** Laura O'Byrne

**Affiliations:** University College Cork, Ireland

**Systematic review protocol: Patient reported outcome measures in childbirth and postpartum maternal quality of life: a protocol for systematic review of measurement properties.**

 Thank you to the third reviewer for taking the time to review the protocol and for their comments.

***Review comment 1:*** *Is the aim of the review to identify tools, or is the aim of the review to evaluate the psychometric properties of tools used to assess various measures of QoL in postpartum women? Is this specific part of the project to develop a new tool, or is this specific part of the project to assess what already exists, to take forward to produce a new tool? The review question should reflect this very specific aim.*

**Response 1**:

 Thank you, this systematic review aimed to both identify the tools available and to evaluate their psychometric properties. The objectives in the abstract read ‘This systematic review aims to identify and evaluate existing patient reported assessment measures/tool(s) that can be used in developing a PROM for postpartum women. We will assess and evaluate their measurement properties in a transparent and structured way in accordance with the COSMIN guidelines.’

 The aim in introduction originally read ‘The aim of this review is to identify potential tool(s)/measurement that can be used to produce a PROM for postpartum women who have been given care within a maternity system’. We have now edited it to read ‘This study aims to identify and evaluate patient reported assessment measures/tool(s) that can be used in developing a PROM for postpartum women. Their measurement properties will be assessed in a transparent and structured way in accordance with the COSMIN guidelines.’

***Reviewer comment 2: **Search strategy: 3 databases is very much the minimum expected for a systematic review. I would generally expect to see more databases searched, and to include the Cochrane library, plus PsychInfo as this is a review of psychometric properties. More supplementary searching should be included, for example, citation searching.*

**Response 2: ** The search strategy states ‘A systematic literature review to examine all available objective tools for patient assessment of QoL and health assessment after hospital based maternity care will be performed using the following bibliographic databases: PubMed, CINHAL and EMBASE. Databases will be searched from inception to present and there will be no language restrictions set. Additionally, hand searching of the reference lists of the studies included and key articles on this topic will be searched.’

 By searching these three databases we were hopeful that any articles on Cochrane or PsycInfo of relevance would be indexed in the above. This search strategy yields over 10,000 studies. Citation search will be included ‘Additionally, hand searching of the reference lists of the studies included and key articles on this topic will be searched.’

***Reviewer comment 3:*** *Inclusion criteria: Much more detail is need here. PICO isn’t appropriate as childbirth isn’t an ‘intervention’. You could include it as an ‘exposure’, but as there is really no comparator - a PEO structure would be more relevant.*

**Response 3:**Thank you – we have changed the inclusion criteria section to read – ‘The eligibility criteria for this review followed the COSMIN guidelines for systematic reviews of PROM recommendations. Eligible studies included generic and specific PROM that assessed postpartum women’s health and wellbeing, where the study sample included >50% postpartum women as advised by COSMIN. The aim of the eligible study should be evaluation of one or more measurement properties of the tool, or the evaluation of the interpretability of the PROMs of interest. Studies that only use the PROM as a measurement instrument for the outcome of interest or in which the PROM was used for the validation of another instrument were excluded. Only full-text articles were included. All peer reviewed studies with an assessment tool designed for patient completion were considered.’

***Reviewer Comment 4:*** *A section on ‘types of study’ would also be informative here. The protocol states that ‘The study should concern PROM and finally the aim should be the evaluation of one or more measurement properties, the development of a prom or the evaluation of the interpretability of the PROM of interest.’ I think further detail is needed on the types of PROM that are of interest.*

**Response 4: **Thank you – we have changed the text in the Eligibility criteria to read – ‘Eligible studies included generic and specific PROM that assessed postpartum women’s health and wellbeing, where the study sample included >50% postpartum women as advised by COSMIN. The aim of the eligible study should be evaluation of one or more measurement properties of the tool, or the evaluation of the interpretability of the PROMs of interest. Studies that only use the PROM as a measurement instrument for the outcome of interest or in which the PROM was used for the validation of another instrument were excluded. Only full-text articles were included. All peer reviewed studies with an assessment tool designed for patient completion were considered.’

***Reviewer Comment 5:*** *The authors have stated in the abstract that there are no maternity specific PROMs. Presumably these will still be searched for however – recent PROMs may have been published. In addition, more clarification is needed on what type of PROMs are expected to be included in light of the anticipated lack of condition-specific PROMs – i.e. generic PROMs such as EQ5D, SF-36 etc. So will the authors be retrieving studies which include the development/validation of generic tools, but only where they have been applied to maternity-specific populations?*

**Response 5: ** Thank you, any tool (generic or condition-specific) examining women’s health and well-being postpartum will be included. The new Eligibility criteria text now reads ‘Eligible studies included generic and specific PROM that assessed postpartum women’s health and wellbeing, where the study sample included >50% postpartum women as advised by COSMIN.’

***Reviewer Comment 6*** *For ‘outcomes’ – the authors state outcome is quality of care. Do they mean quality of life? However, isn’t the outcome the psychometric properties of the tools that are being used to assess QoL? These properties as described in the COSMIN methodology should be briefly listed e.g. content validity; structural validity; internal consistency etc.*

**Response 6:**Thank you – we have changed the inclusion criteria section to read – ‘Eligible studies included generic and specific PROM that assessed postpartum women’s health and wellbeing, where the study sample included >50% postpartum women as advised by COSMIN.’

 The data extraction section reads ‘The data to be extracted as recommended by the COSMIN systematic review guidelines include the characteristics of the tool, characteristics about the population assessed, results on the measurement properties’ We have now added ‘which includes: structural validity, internal consistency, cross-cultural validity/measurement invariance, reliability, measurement error, criterion validity, hypotheses testing for construct validity, and responsiveness.’

***Reviewer Comment 7:**Finally, will the COSMIN ratings be ratified by subject experts and patients at the end of the process?*

**Response 7:** Thank you. The review will be conducted by subject matter experts, including obstetricians and gynaecologists actively involved in providing care at a tertiary-level maternity hospital and authors who have experience in designing and implementing PROM. The results of this systematic review will be Piloted by patients to better comprehend the recommended tools' content and validity, but patients will not be involved in the systematic review itself.

# Reviewer report: Reviewer response for version 2

**DOI:** 10.21956/hrbopenres.14957.r33331

**Published:** 2023-01-16

**Authors:** Fiona Dickinson

**Affiliations:** 1 Liverpool School of Tropical Medicine, Liverpool, UK

No new comments.

Is the study design appropriate for the research question?

Yes

Is the rationale for, and objectives of, the study clearly described?

Yes

Are sufficient details of the methods provided to allow replication by others?

Yes

Are the datasets clearly presented in a useable and accessible format?

Yes

Reviewer Expertise:

Development of PROMs for use in maternity services in low and middle-income countries; Emergency obstetric care in LMIC; Care of mothers and newborns in LMIC.

I confirm that I have read this submission and believe that I have an appropriate level of expertise to confirm that it is of an acceptable scientific standard.

# Reviewer report: Reviewer response for version 1

**DOI:** 10.21956/hrbopenres.14653.r32819

**Published:** 2022-09-23

**Authors:** Christine Newman

**Affiliations:** 1 National University of Ireland, Galway, Ireland

This is an interesting and novel piece which aims to review existing PROMs and patient reported assessment measures and use this to develop a PROM for the post-partum period.

 There are some small spelling errors that can be addressed through proofreading.

 Only 2 databases will be searched - at least 3 are generally advised for a systematic review. Can the authors comment on this?

 There is no sample search strategy given keywords, MESH headings, etc. This should be included in the protocol.

Is the study design appropriate for the research question?

Yes

Is the rationale for, and objectives of, the study clearly described?

Yes

Are sufficient details of the methods provided to allow replication by others?

Partly

Are the datasets clearly presented in a useable and accessible format?

Yes

Reviewer Expertise:

Diabetes in pregnancy and Patient-Reported Outcomes

I confirm that I have read this submission and believe that I have an appropriate level of expertise to confirm that it is of an acceptable scientific standard, however I have significant reservations, as outlined above.

# Response

**Published:** 2023-01-10

**Authors:** Laura O'Byrne

**Affiliations:** University College Cork, Ireland

Thank you to the two reviewers for taking the time to review this protocol and for their comments.

**Reviewer 2, comment 1** *‘* *Only 2 databases will be searched - at least 3 are generally advised for a systematic review. Can the authors comment on this?’*

**Response:** Thank you, we increased the search strategy to include searching on the CINAHL database. The search, therefore, included three databases PubMed, EMBASE and CINAHL which yielded 10,324 studies identified in the initial search, of which 29 tools were identified from 41 eligible studies included in the review. This has been added to the manuscript search strategy line 52 ‘A systematic literature review to examine all available objective tools for patient assessment of QoL and health assessment after hospital-based maternity care will be performed using the following bibliographic databases: PubMed , CINHAL and EMBASE.’  The Abstract methods section has also been amended to include ‘Methodological guidelines for systematic reviews of PROMs have been developed by the COSMIN initiative and will be followed for this systematic review. A systematic literature review will be performed using PubMed, CINAHL and EMBASE from inception to the present day.’

**Reviewer 2, comment 2**: *‘* *There is no sample search strategy given keywords, MESH headings, etc. This should be included in the protocol.’* 

**Response:** The search strategy is included in the extended data held within the Harvard Dataverse: Search thread: Patient reported outcome measures in childbirth and postpartum maternal quality of life: a protocol for the systematic review of measurement properties. https://doi.org/10.7910/DVN/WMHKXG 16.

 We thank you for your helpful comments that have improved the quality of the paper and thank you for re-considering this manuscript for review and publication.

# Reviewer report: Reviewer response for version 1

**DOI:** 10.21956/hrbopenres.14653.r32545

**Published:** 2022-08-18

**Authors:** Fiona Dickinson

**Affiliations:** 1 Liverpool School of Tropical Medicine, Liverpool, UK

Thank you for this interesting and timely paper describing your systematic review protocol, to identify and evaluate PROMs for postnatal women. On the whole, the protocol seems appropriate and achievable. The rationale and objectives are clearly described, the proposed study design seems appropriate, and the proposed methods are described in detail.

 There are however, a few typos and it would be good to be consistent in the use of the acronym PROM/S/s. Also, the 3 paragraphs under the headings 'Data extraction' and 'Generating recommendations for the use of a PROM', seem to be a direct repeat. Good luck with the review and I look forward to seeing the results.

Is the study design appropriate for the research question?

Yes

Is the rationale for, and objectives of, the study clearly described?

Yes

Are sufficient details of the methods provided to allow replication by others?

Yes

Are the datasets clearly presented in a useable and accessible format?

Yes

Reviewer Expertise:

Development of PROMs for use in maternity services in low and middle-income countries; Emergency obstetric care in LMIC; Care of mothers and newborns in LMIC.

I confirm that I have read this submission and believe that I have an appropriate level of expertise to confirm that it is of an acceptable scientific standard.
